# Pt/CeO_2_ as Catalyst for Nonoxidative Coupling of Methane: Oxidative Regeneration

**DOI:** 10.1021/acs.jpclett.3c01179

**Published:** 2023-07-21

**Authors:** Hao Zhang, Valery Muravev, Liang Liu, Anna Liutkova, Jérôme F. M. Simons, Blanka Detlefs, Huaizhou Yang, Nikolay Kosinov, Emiel J. M. Hensen

**Affiliations:** † Laboratory of Inorganic Materials and Catalysis, Department of Chemical Engineering and Chemistry, Eindhoven University of Technology, 5600 MB Eindhoven, The Netherlands; ‡ European Synchrotron Radiation Facility, 71 avenue des Martyrs, CS 40220, 38043 Grenoble, France

## Abstract

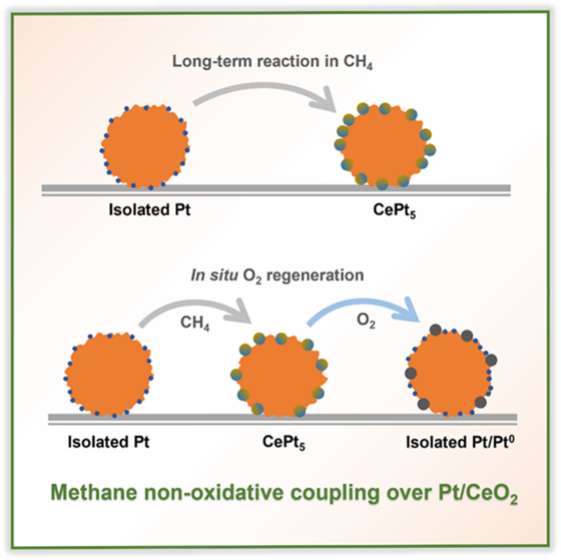

Direct nonoxidative coupling is a promising route for methane upgrading, yet its commercialization is hindered by the lack of efficient catalysts. Pt/CeO_2_ catalysts with isolated Pt species have attracted an increasing amount of interest in recent years. Herein, we studied the catalytic role and evolution of isolated Pt centers on CeO_2_ prepared by flame spray pyrolysis under the harsh reaction conditions of nonoxidative methane coupling. During the reaction at 800 °C, the isolated Pt sites sinter, leading to a loss of the ethylene and ethane yield. The agglomerated Pt can be redispersed by using an *in situ* regeneration strategy in oxygen. We found that isolated Pt centers are able to activate methane only at the initial reaction stage, and the CePt_5_ alloy acts as the active phase in the prolonged reaction.

Nonoxidative coupling of methane to more valuable hydrocarbons is a potential route for natural gas and biogas valorization.^1−4^ The pioneering study of a highly active and stable Fe©SiO_2_ catalyst by Bao and co-workers led to a substantial research effort in this area.^1,5,6^ The isolated Fe centers in the Fe©SiO_2_ catalyst are argued to limit C–C coupling and, therefore, coke formation on the catalytic surface. Based on this insight, other catalysts containing isolated metal sites such as Pt/CeO_2_,^7−11^ Pt/C_3_N_4_,^12^ and Ru/TiO_2_^13^ have been explored for nonoxidative coupling of methane. Xie et al. were the first to report on the use of Pt/CeO_2_ with isolated Pt sites for the NOCM reaction.^7^ A methane conversion of 14.4% with a C_2_-hydrocarbon selectivity of 74.6% was obtained from a 1 vol % CH_4_ feed. The authors reported that Pt remained isolated during a 40 h stability test. Later, Bajec et al. used microkinetic analysis of Pt/CeO_2_ catalysts with isolated Pt sites to demonstrate that hydrogen abstraction from methane determines the overall rate, while surface C–C coupling reactions only slightly affect the overall CH_4_ conversion.^8^ Eggart et al. investigated Pt-doped CeO_2_ catalysts by *operando* X-ray absorption spectroscopy and synchrotron-based vacuum ultraviolet photoionization mass spectrometry.^11^ Methyl radicals were detected as the main reaction intermediate, indicating that gas-phase reactions are involved in the formation of C_2_-hydrocarbons. Different from the study by Xie et al.,^7^ these authors observed extensive sintering of the initially isolated Pt sites. The agglomeration of Pt was observed even after He treatment at this temperature. The authors also contended that methane activation can occur at the interface between Pt and the ceria support. Theoretical studies have also been carried out to understand the reaction mechanism on such Pt/CeO_2_ catalysts. Wen et al. performed a detailed density functional theory (DFT) study, showing that methane conversion strongly depends on the Pt particle size.^9^ Isolated Pt on CeO_2_ was predicted to exhibit the highest C_2_-hydrocarbon selectivity. Chang and co-workers reported that the active sites in Pt/CeO_2_ involve two functionalities, namely, single Pt atoms close to a frustrated Lewis acid obtained upon oxygen removal from ceria, which are together involved in methane activation under NOCM conditions.^10^ Despite the growing interest in Pt/CeO_2_ for the NOCM reaction, it remains unclear how isolated Pt sites behave under harsh reaction conditions.

In the present study, Pt/CeO_2_ was prepared by the one-step flame spray pyrolysis (FSP), which is a proven method to synthesize catalysts with highly dispersed metal species.^11,14^ Fourier and wavelet transformed EXAFS demonstrates the isolated nature of Pt in which only one clear Pt–O coordination shell at ∼1.6 Å is observed (Figure 1a, Figure S1). XPS analysis shows the predominance of Pt^2+^ species, with a Pt 4f_7/2_ binding energy of ∼72.9 eV on the surface of the fresh catalyst (Figure S2). Previous experimental and theoretical studies have supported the interpretation of such a Pt feature in terms of isolated Pt^2+^ ions in a square planar configuration in the CeO_2_ surface.^15−18^ The main products of the NOCM reaction with the Pt/CeO_2_ catalyst at a reaction temperature of 800 °C were ethylene and ethane (Figure 1b). At the given conditions, ethane is obtained in larger amounts than ethylene. Coke was also formed on the Pt/CeO_2_ catalyst (Figure S3). During the initial stage of reaction, the methane conversion decreases from ∼1% to ∼0.2%, which goes together with a slow increase of the ethane and ethylene yield. The Pt/CeO_2_ catalyst exhibits a C_2_-hydrocarbon selectivity of 68% at a methane conversion rate of 0.85 mmol_CH4_ g_Pt_^–1^ min^–1^, which is comparable with earlier reported activity data for Pt/CeO_2_ tested under similar reaction conditions, namely, a C_2_-hydrocarbon selectivity of 55% at a reaction rate of 0.34 mmol_CH4_ g_Pt_^–1^ min^–1^ at 780 °C.^8^ The reaction involves an induction period, which is probably caused by the transformation of the initially isolated Pt^2+^ species. We characterized in detail the fresh and used Pt/CeO_2_ catalysts. The synchrotron XRD pattern of fresh Pt/CeO_2_ is dominated by diffraction lines from CeO_2_ (PDF no. 00-004-0593). The pattern of the used sample contains additional features due to Ce_2_O_3_ (PDF no. 01-083-5456) and CePt_5_ alloy (PDF no. 01-071-7052, Figure 1c, Figure S4). Diffraction peaks of Pt metal (PDF no. 00-004-0802) were not observed. The formation of CePt_5_ in a Pt/CeO_2_ sample reduced at high temperature in H_2_ or CH_4_ has been reported before.^11,19^ Rietveld refinement points to the sintering of CeO_2_ particles during the NOCM reaction (Figure S5 and Table S1), which is confirmed by TEM (Figure S6). The formation of CePt_5_ is in line with XANES and EXAFS analysis (Figure 1a, Figures S1, S7, and S8, and Table S2). The reduction of Pt is also in keeping with the absence of Pt^2+^ in the XPS result of the used catalyst (Figure S2). Therefore, we can conclude that Pt on the catalyst surface was fully reduced into CePt_5_. As such, it can be stated that the CePt_5_ alloy is the active phase during a prolonged NOCM reaction, although the role of the initially present atomically dispersed Pt^2+^ sites needs to be further investigated.

**Figure 1:**
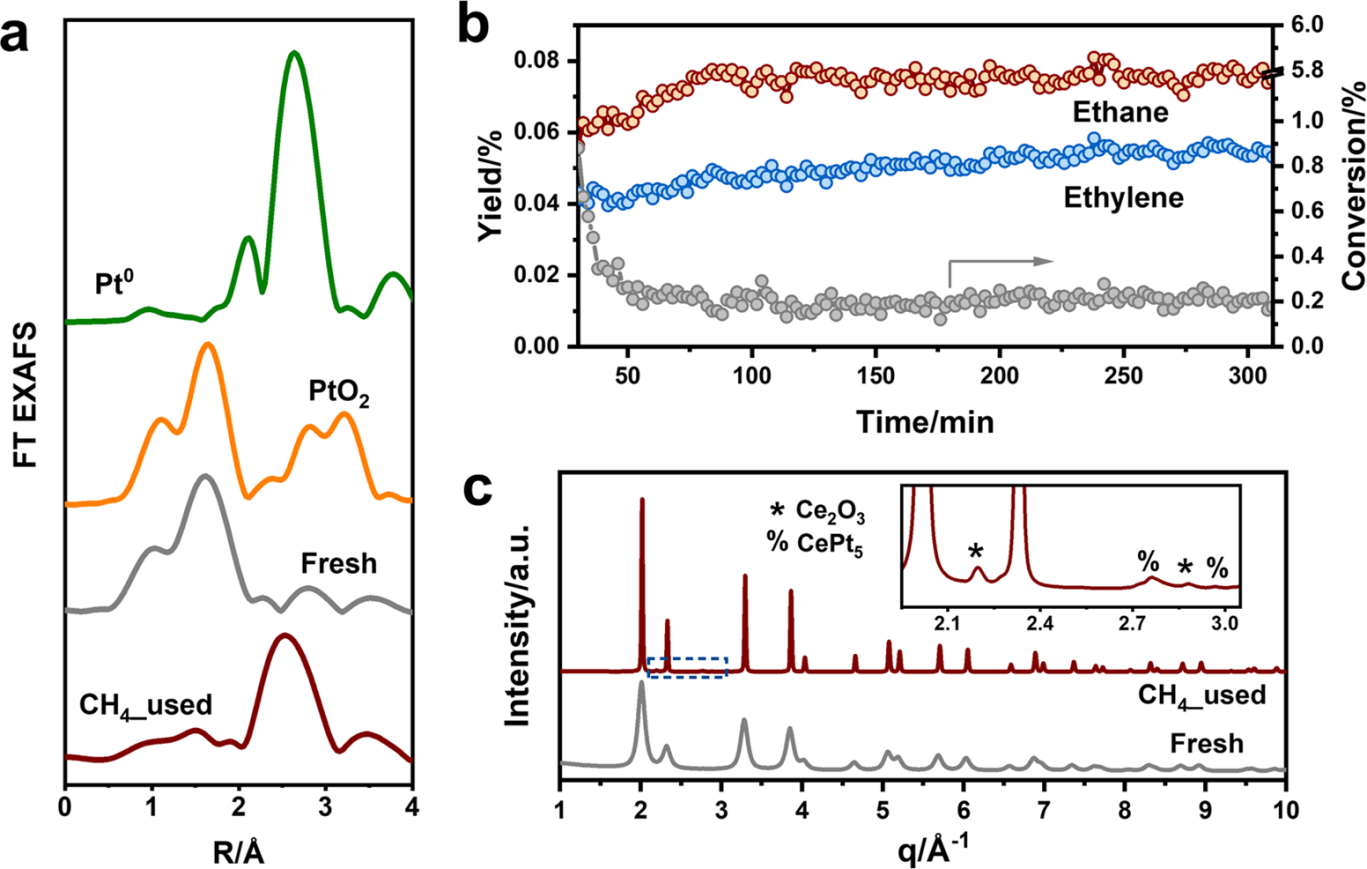
(a) *k*^3^-weighted EXAFS measured at the Pt L_3_ edge of fresh and used Pt/CeO_2_ catalysts. The Pt^0^ and PtO_2_ references are included for comparison. (b) Catalytic performance of the Pt/CeO_2_ catalyst at 800 °C using 100 mg catalysts under 10 mL/min 95 vol % CH_4_ with 5 vol % Ar. (c) Synchrotron XRD patterns of fresh and used Pt/CeO_2_ catalysts. The inset shows the enlarged region highlighted by the blue dashed cube.

We developed an oxidative regeneration strategy based on the work of Datye and co-workers. They reported that gaseous PtO_2_ can be trapped by CeO_2_, leading to isolated Pt species.^20^ We employed an *in situ* O_2_ regeneration at high temperature, consisting of periodically switching between a CH_4_-containing reaction feed and an O_2_-containing regeneration feed, aiming at the redispersion of agglomerated Pt. The evolution of the MS signals of 27 (ethylene and ethane) and 30 (ethane) in Figure 2a during consecutive reaction–regeneration cycles indicates the rapid decline of the C_2_-hydrocarbon yield after the initial period (i.e., the *m*/*z* = 27 signal drops by 1 order of magnitude). This deactivation is likely caused by the rapid sintering of Pt in Pt/CeO_2_. By optimizing the length of the reaction and regeneration treatments, we found that a regeneration period of 10 min in O_2_ can already restore the initial catalytic activity. Nevertheless, the reaction–regeneration cycles lead to a decrease of the maximum C_2_-hydrocarbon signals at *m*/*z* = 27 by nearly an order of magnitude after 4 cycles (Figure 2a). The higher formation rate of C_2_-hydrocarbons upon regeneration was confirmed by GC analysis (Figure 2b). Other products including H_2_, H_2_O, and CO_2_ and small amounts of benzene and toluene were also detected (Figure S9). To confirm the pivotal role of Pt, we also studied the reaction–regeneration cycles for CeO_2_. Without Pt, the C_2_-hydrocarbon signal at *m*/*z* = 27 is about 3 times lower in the first reaction cycle (Figure S10). The hydrocarbon products obtained with the bare CeO_2_ support are likely due to homogeneous gas-phase reactions. Nevertheless, we cannot completely rule out the catalytic role of CeO_2_ in methane activation. Chang and co-workers reported the reduced CeO_2_ surface to be active in the NOCM reaction. Based on theoretical modeling, they proposed that frustrated Lewis pairs, involving surface Ce cations and oxygen vacancies, are candidate active sites for methane activation.^21^ Nevertheless, the catalytic activity is substantially enhanced when Pt is introduced, showing that Pt plays an important role in the catalytic activity of Pt/CeO_2_ catalysts. This conclusion is also in line with other studies of the role of Pt in Pt/CeO_2_ for the NOCM reaction.^7,11^

**Figure 2:**
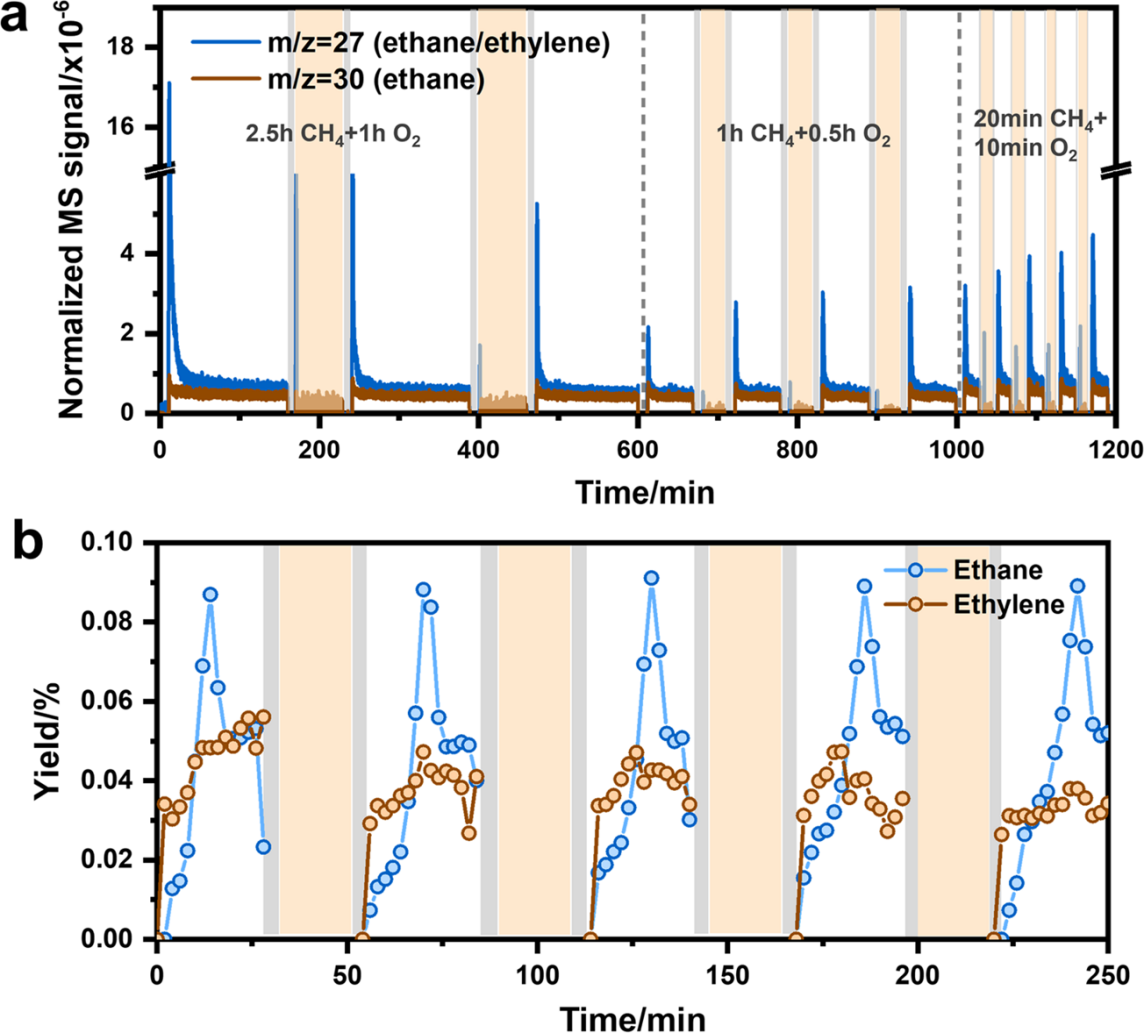
(a) Reaction–regeneration tests of the Pt/CeO_2_ catalyst at 800 °C using 100 mg catalysts. A flow of 50 mL/min of He was purged to the reactor for 10 min between the NOCM reaction in 10 mL/min of 95 vol % CH_4_ and the regeneration under 40 vol % O_2_. (b) The ethane and ethylene yield obtained by GC during the reaction–regeneration cycles using 100 mg of Pt/CeO_2_ catalyst at 800 °C. A total flow of 10 mL/min (95 vol % CH_4_ + 5 vol % Ar for reaction and 40 vol % O_2_ diluted by He for regeneration) was used for the measurements. The frequency of GC injection is every 2 min. The gray zone indicates the He purging, and the orange zone represents the regeneration treatment in O_2_.

The Pt/CeO_2_ sample was characterized in detail after each reaction and regeneration cycle. The formation of Ce_2_O_3_ upon an NOCM cycle follows from the XRD patterns of CH_4__1 and CH_4__3 (in CH_4__n, “n” refers to the nth reaction cycle) and is likely caused by the reduction of CeO_2_ by CH_4_. However, the main phase present in the used materials is CeO_2_. Ce_2_O_3_ was oxidized during regeneration as follows from the XRD patterns of the corresponding regenerated catalysts O_2__1 and O_2__3 (Figure 3a, in O_2__n, “n” refers to the nth regeneration cycle). With respect to Pt, the diffraction line at ∼2.79 Å^–1^ can be linked to the Pt metal in the O_2__1 and the O_2__3 samples. Upon reaction in CH_4_, diffraction lines of CePt_5_ are observed. Rietveld refinement of the synchrotron XRD patterns indicates the sintering of CeO_2_ from ∼7 to ∼120 nm during the three reaction–regeneration cycles. The sintering of CeO_2_ slowed after the third reaction stage. Meanwhile, the crystallite size of the CePt_5_ particles increased from ∼4 to ∼8 nm, while the Pt^0^ species observed after the oxidative regeneration have a size of ∼20 nm (Table S1 and Figure S5). We further studied these samples by Raman spectroscopy (Figure 3b). The Raman bands at ∼550 and ∼690 cm^–1^, which can be clearly observed in the fresh Pt/CeO_2_ catalyst, are due to the asymmetric and symmetric stretching modes of the Pt–O–Ce moiety, respectively.^22^ These two bands are not present anymore after the NOCM reaction but reappeared upon regeneration in O_2_ albeit with a weaker intensity. The Raman results can be explained by the aggregation of Pt during the NOCM reaction and, subsequent, Pt redispersion during regeneration in the presence of O_2_. XPS analysis reveals that 75% of surface Pt species are present in the reduced state after the first reaction cycle with the remaining 25% in the Pt^2+^ state. The fraction of Pt^2+^ increased to 88% after O_2_ regeneration, pointing to redispersion of Pt on the catalyst surface (Figure 3c and Table S3). TEM analysis of Pt/CeO_2_ catalysts also suggests the agglomeration and partial redispersion of Pt species upon CH_4_ and O_2_ treatments (Figure S11).

**Figure 3:**
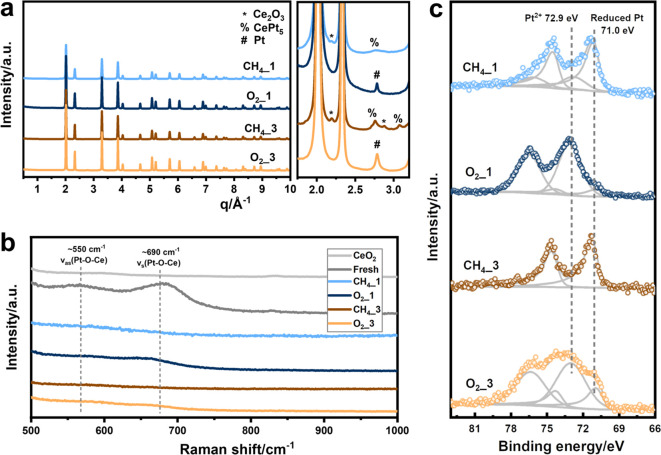
(a) Quasi *in situ* synchrotron XRD patterns with the enlarged region. (b) Raman spectra measured using the 532 nm laser. (c) Pt 4f XPS results of Pt/CeO_2_ catalysts at different reaction/regeneration stages. In CH_4__n and O_2__n, “n” refers to the nth reaction or regeneration. CH_4__1 indicates the catalyst obtained after the first reaction stage, and the corresponding O_2__1 suggests the regenerated CH_4__1 sample after the O_2_ treatment.

Quasi *in situ* EXAFS analysis was carried out to follow the changes in the Pt phase (Figures S12 and S13). The Pt–O coordination shell at ∼1.6 Å disappeared after the NOCM reaction in CH_4_. However, this shell can be seen again after O_2_ regeneration. There is also a second shell in the EXAFS after CH_4_ or O_2_ treatment, which can be linked to the formation of CePt_5_ and/or Pt^0^ particles based on the above-discussed synchrotron XRD data. As the second shell after the regeneration in O_2_ cannot be assigned to bulk PtO_2_ or PtO particles based on reference compounds, the presence of a Pt–O shell can be related to the partial redispersion of sintered Pt (Figure S12). EXAFS fitting shows that the coordination number of the Pt–O shell decreased from ∼3.8 to ∼0.4 during the first NOCM reaction and increased again to ∼2.8 during the first O_2_ regeneration step. The coordination number of the Pt–O shell obtained after the third O_2_ regeneration was lower at a value of ∼1, indicating a lower extent of redispersion (Figure 4a, Figure S14, and Table S4). Based on the above analysis, we infer that the enhanced catalytic performance of the Pt/CeO_2_ catalyst after O_2_ regeneration is caused by the reappearance of isolated Pt centers.

**Figure 4:**
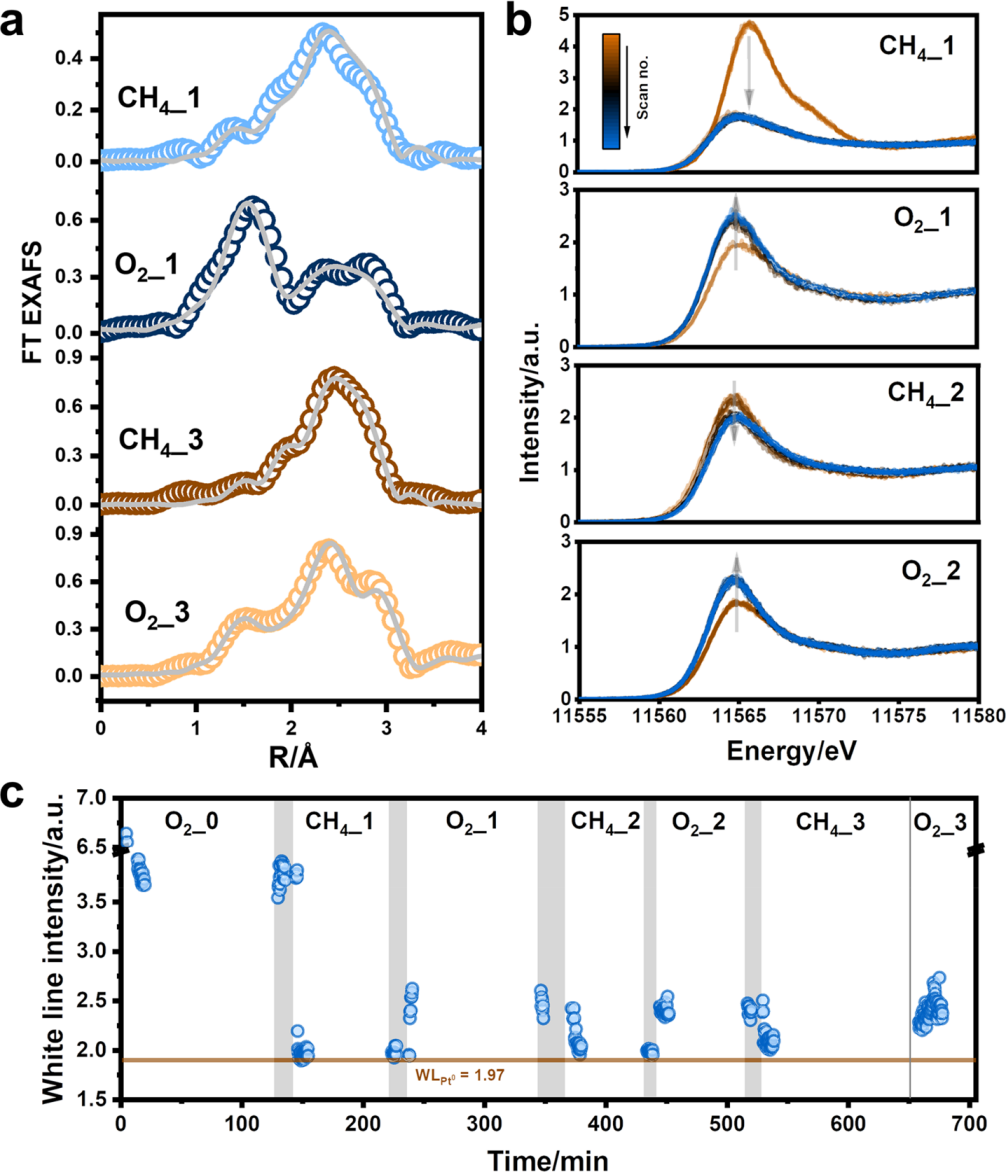
(a) Quasi *in situ k*^2^-weighted EXAFS at the Pt L_3_-edge of the Pt/CeO_2_ catalyst at different reaction and regeneration stages. The raw data are shown using open circles, and the gray lines indicate the fitting results. (b) Pt L_3_-edge HERFD-XANES spectra of the Pt/CeO_2_ catalyst at different reaction and regeneration stages. (c) The evolution of white line intensity during the *in situ* HERFD-XANES measurements at the Pt L_3_-edge. The measurements were performed at 800 °C using 30 mg of Pt/CeO_2_ catalysts under 5 mL/min CH_4_ for reaction and 15 mL/min 40 vol % of O_2_ for regeneration. A flow of 20 mL/min of He was purged to the *in situ* cell between the NOCM reaction and the catalyst regeneration. The position of the white line intensity of Pt^0^ is labeled. The He-purging period is labeled in gray.

According to XPS and EXAFS, the reoxidation of reduced Pt to Pt^2+^ occurs during the redispersion of the sintered Pt species. Therefore, the redispersion of Pt can be tracked by following the oxidation state of Pt via the white line intensity of the Pt L_3_-edge (Figure S15).^23^ For this purpose, we carried out *in situ* HERFD-XAS to confirm the Pt evolution under actual reaction conditions. HERFD-XANES can provide enhanced energy resolution compared with the conventional total fluorescence yield detection method, allowing better monitoring of the changes in the XANES region (Figure S16). A home-built high-temperature *in situ* XAS cell with quartz tube reactor was used for the HERFD-XANES experiments at 800 °C (Figures S17 and S18). The white line intensity recorded at the Pt L_3_-edge was employed to investigate the evolution of Pt (Figure 4b,c).^23^ Upon heating the fresh catalyst in 40 vol % of the O_2_ to 800 °C, the white line intensity decreased, indicating the reduction of Pt (Figure S19). After switching to CH_4_, Pt reduction proceeded, as revealed by the further decrease of the white line intensity. This reflects the sintering of Pt. The Pt species were reoxidized after exposure to O_2_, which implies redispersion of Pt centers. We continued the reaction–regeneration experiments and observed the periodic reduction and oxidation of Pt in CH_4_ and O_2_, respectively (Figure 4c). We compared the HERFD-XANES spectra obtained after each reaction and regeneration stage with the spectra of Pt^0^ and CePt_5_. The HERFD-XANES after the NOCM reaction is closer to that of CePt_5_, whereas the O_2_ regeneration step induced the formation of Pt^0^ (Figure S20). These findings are consistent with the synchrotron XRD results. To determine the structure of the Pt phase, we measured HERFD-EXAFS of the Pt/CeO_2_ catalyst at different reaction and regeneration stages at room temperature (Figure S21). It is clear that an additional shell at ∼1.6 Å appears after the O_2_ treatment, which can be ascribed to the Pt–O single scattering path. These *in situ* tests confirm the redispersion of Pt after the O_2_ regeneration. We verified that X-ray beam damage of the samples was negligible during the *in situ* HERFD-XAS measurements (Figure S22).

To summarize, Pt/CeO_2_ with isolated Pt sites was investigated as a catalyst for the methane nonoxidative coupling reaction. Sintering of the initially isolated Pt^2+^ sites in CeO_2_ is inevitable, involving the reduction not only of Pt but also of part of Ce, leading to CePt_5_ particles. This CePt_5_ alloy acts as the active phase during the prolonged NOCM reaction. An *in situ* O_2_ regeneration strategy was developed, in which the Pt-containing particles can partially redisperse. This work highlights the dynamic nature of the Pt species in Pt/CeO_2_ during the NOCM reaction and oxidative regeneration.

## Supporting Information Available

Experimental details; EXAFS, XANES, Raman, and XPS results of fresh and used Pt/CeO_2_ catalysts and the catalyst after different reaction or regeneration stages; crystal structure; Rietveld refinement results of synchrotron XRD patterns; TEM images of Pt/CeO_2_ catalysts; the results of the switching experiments over CeO_2_; EXAFS fitting results of Pt/CeO_2_ catalysts; MS data; surface fraction data; the procedure, experimental setup, and results of *in situ* HERFD-XANES measurements (PDF)
