# Supplementary material for: Pt/CeO2 as Catalyst for Nonoxidative Coupling of Methane: Oxidative Regeneration
Source: J Phys Chem Lett. 2023 Jul 21;14(30):6778–83. doi: 10.1021/acs.jpclett.3c01179 (PMC10405266; doi:10.1021/acs.jpclett.3c01179)
Supplement: Supplementary file 1 — jz3c01179_si_001.pdf [file jz3c01179_si_001.pdf]

Supporting Information

Pt/CeO_2 as Catalyst for Nonoxidative Coupling of Methane: Oxidative Regeneration

Hao Zhang,[1] Valery Muravev,[1] Liang Liu,[1] Anna Liutkova,[1] Jérôme F. M. Simons,[1] Blanka Detlefs,[2] Huaizhou Yang,[1] Nikolay Kosinov,[1,] and Emiel J. M. Hensen[1,*]*

[1]Laboratory of Inorganic Materials and Catalysis, Department of Chemical Engineering and Chemistry, Eindhoven University of Technology, 5600 MB Eindhoven, The Netherlands

[2]European Synchrotron Radiation Facility, 71 avenue des Martyrs, CS 40220, 38043 Grenoble, France

Corresponding authors

n.a.kosinov@tue.nl (N. Kosinov); e.j.m.hensen@tue.nl (E.J.M. Hensen)

Experimental details

Synthesis of Pt/CeO_2 catalysts

Flame spray pyrolysis was employed to synthesize the Pt/CeO_2. In a typical procedure, desired amounts of platinum(II) acetylacetonate (Sigma Aldrich) and cerium(III) acetylacetonate (Sigma Aldrich) were dissolved in a 1:1 (vol.%) solvent mixture of acetic acid (Alfa Aesar) and 2-ethylhexanoic acid (Sigma Aldrich) at ~50 °C. The Ce concentration was 0.15 M, while the Pt concentration was adjusted to a target Pt content of 1 wt.%. The resulting solution was injected to the nozzle of the FSP setup (TETHIS NPS10) with a flow rate of 5 mL/min maintained by a syringe pump. Methane (1.5 L/min) and oxygen (3 L/min) were fed into the FSP setup to generate a flame, using an oxygen flow of 5 L/min oxygen as dispersion gas. The as-prepared sample was collected using a glass fiber filter placed inside the FSP setup. Based on elemental analysis using inductively coupled plasma-optical emission spectroscopy (ICP-OES), the actual Pt loading was determined to be 1.09 wt.%. The as-prepared Pt/CeO_2 sample will be further denoted as the fresh catalyst. A CeO_2 reference sample was prepared in the same way without Pt in the solution.

Catalyst characterization

Transmission electron microscopy (TEM). A FEI Tecnai Sphera (200 kV acceleration voltage) instrument equipped with the LaB_6 filament was used to study the morphology of Pt/CeO_2 catalysts at different reaction and regeneration stages. A specific amount of catalyst powder was dispersed in ethanol under ultrasonic treatment. After that, the dispersion was dropped on a Cu grid, coated with holey carbon film (Quantifoil), and dried in air at room temperature.

X-ray photoelectron spectroscopy (XPS). XPS measurements were conducted using a Thermo Scientific *K*-Alpha XPS instrument with aluminum anode (Al $K\alpha$ = 1486.68 eV). The

powder samples were placed on a double-sided carbon tape (SPI supplies) for measurements. CasaXPS software (version 2.3.23) was used to analyze the XPS spectra. The U''' component of the Ce *3d* core line (916.7 eV) was used for energy calibration.[1] A Shirley background was applied. Pt^{2+} was fitted using a symmetric function using the GL(30) line shape, while metallic Pt species were fitted using an asymmetric function (line shape LF(0.56, 1.5, 55, 150)).[1]

Inductively coupled plasma-optical emission spectroscopy (ICP-OES). ICP-OES (Ametek SPECTROBLUE EOP) was used to determine the Pt loading in the Pt/CeO_2 catalyst. The sample was digested in a mixture of 4 mL of diluted H_2SO_4 (98 % H_2SO_4 : H_2O = 1:1) and 2 mL of 65 % HNO_3 under microwave treatment (Multiwave 7000, Anton Paar) at 140 bar and 280 °C for 1.5 h.

Raman spectroscopy. Raman spectroscopy was performed using a WITec WMT50 confocal Raman microscope with a WITec UHTS300 spectrometer. The spectra were directly collected over powder samples using a 532 nm laser with the power of 5 mW.

X-ray diffraction (XRD). Quasi *in situ* synchrotron XRD measurements were performed at the ID31 beamline of European Synchrotron Radiation Facility (ESRF, located at Grenoble, France). The tests were carried out at 75 keV in transmission mode. A Dectris Pilatus 3X CdTe 2M detector was used to collect the scattered signal. The treated samples were sealed in a 3 mm o.d. Kapton tubes inside a glovebox. Beeswax (Fisher Scientific) was used to seal the two ends of the tube. The detector distance, energy, and tilts were calibrated using the NIST CeO_2 powder. The obtained 2D patterns were integrated using the pyFAI package.[2] Rietveld refinement analysis was carried out using the GSAS II software (version 5000).[3]

X-ray absorption spectroscopy (XAS). Quasi *in situ* XAS measurements at the Pt L_3-edge were carried out at the CLÆSS beamline of the ALBA synchrotron (Barcelona, Spain) using a Si(111) double-crystal monochromator. The samples were pressed into self-standing pellets and sealed with Kapton tape inside a glovebox. The Pt amount in each pellet was below 0.5 wt.%.

The samples were analyzed at room temperature and a total pressure of ~0.07 mbar. A 4-element silicon drift detector was used to record the XAS spectra in fluorescence mode. The XAS spectra were collected in a continuous scanning mode, and the resulting 41 spectra for each sample were merged to increase the signal to noise ratio. The Pt foil measured simultaneously in transmission mode was employed for energy calibration. The spectra of reference samples were recorded in transmission mode under the same conditions. The X-ray beam size was ~600×600 μm (H×V). The XAS spectrum of the fresh catalyst was collected at the ROCK beamline of SOLEIL (Gif-sur-Yvette, France) using a cryogenically cooled Si(111) channel-cut quick XAS monochromator. The XAS spectra were recorded in fluorescence mode using a PIPS detector. XAS data reduction was carried out using Larch.[4] The FEFF8L code used inside Larch was used to calculate the scattering paths. The amplitude reduction factor (S_0^2) was obtained by fitting the Pt foil reference measured under the same conditions. The change of distance (ΔR), coordination number (CN), energy shift (ΔE_0), and Debye-Waller factor (σ^2) were fitted. Fits were carried out simultaneously in R-space on k^1-, k^2-, and k^3-weighted spectra. The Fourier-transformed EXAFS was plotted without phase-correction. The k^3-weighted EXAFS spectra were used in wavelet transform analysis using a Morlet mother function with the wtEXAFS software package. Pt L_3-edge XANES were simulated using the FEFF10 software.[5, 6] The Hedin-Lundqvist potential was chosen, while simulations were done with the self-consistent field method. The "COREHOLE None" option was applied.[7] The exchange card was set to "0 0 -1.5 -1" in order to suppress core-hole broadening, as high-energy resolution fluorescence detection X-ray absorption near edge structure (HERFD-XANES) was simulated. It was set to "0 0 0 -1" when simulating XANES collected in total fluorescence yield mode.[8]

High energy resolution fluorescence detection X-ray absorption near edge structure (HERFD-XANES). Pt L_3-edge *in situ* HERFD-XANES measurements were recorded at the

ID26 beamline of the European Synchrotron Radiation Facility (ESRF, located at Grenoble, France). A cryogenically cooled Si(111) double-crystal monochromator was employed to select the photon energy. The emission spectrometer was equipped with five spherically bent Ge(660) analyzer crystals installed in a Rowland circle geometry with a radius of 1 m. The signal obtained by the avalanche photodiode detector was normalized to the incident photon flux. The latter is measured by a photodiode recording backscattering from a thin Kapton foil mounted in transmission just upstream of the sample. The HERFD-XANES was measured by detecting the fluorescence signal at the maximum of the Pt $L_{\alpha 1}$ emission line. PyMCA and Larch were used for data reduction.[4, 9] The X-ray beam size was adjusted to ~150×50 μm (H×V). A home-built high temperature *in situ* XAS cell was used in the Huber stage for the *in situ* HERFD-XANES measurements. The time resolution for HERFD-XANES measurements was 60 s. An amount of 30 mg of sieved catalyst (125–250 μm) was placed without dilution in a quartz tube reactor (4 mm i.d., 5 mm o.d. with a 20 mm flattened "window", the thickness of "window" is ~0.2 mm). The sample was fixed by two plugs of quartz wool. The catalyst was heated from room temperature to 800 °C at a rate of 15 °C/min under a 15 mL/min of 40 vol.% O_2/He flow. After reaching 800 °C, the HERFD-XANES measurements were continued during switching experiments. HERFD-EXAFS spectra were also collected at each reaction/regeneration stage. The reaction was carried out in a flow of 5 mL/min CH_4, while regeneration was performed using a 15 mL/min flow of 40 vol.% O_2/He. A He flow of 20 mL/min was used for purging between the reaction and regeneration processes.

Catalytic testing

The catalytic performance of the samples was evaluated in a home-built high temperature fixed-bed reactor operated at 800 °C. The 4 mm i.d. quartz tube reactor was sealed by two Viton O-rings at each end. In a typical experiment, 100 mg of catalysts sieved to 125–250 μm was loaded

into the quartz reactor and fixed by two quartz wool plugs inside the isothermal zone. A total flow of 10 mL/min (95 vol.% CH_4 with 5 vol.% Ar for reaction, and 40 vol.% O_2 diluted by He for regeneration) was used. In the reaction-regeneration tests, the temperature was increased to 800 °C from room temperature using a rate of 10 °C/min in a flow of 10 mL/min of 40 vol.% O_2 followed by an isothermal dwell of 30 min before the reaction-regeneration experiments. A flow of 50 mL/min He was purged to the reactor between the reaction and regeneration processes. For long-term stability evaluation, the temperature was increased from room temperature to 800 °C at a rate of 10 °C/min in a flow of 10 mL/min containing 95 vol.% CH_4 and 5 vol.% Ar, followed by an isothermal dwell of 300 min. A quadrupole mass spectrometer (Balzers Prisma) was used for fast product analysis, while a gas chromatograph (CompactGC 4.0, Interscience) was employed to validate the product formation at an injection interval of 2 min. The Pt/CeO_2 samples retrieved from the reactor after different reaction and regeneration stages were prepared for characterization. The resulting catalysts are denoted "CH_4_n" and "O_2_n", in which "n" indicates the nth reaction or regeneration. For example, CH_4_1 refers to the catalyst after the first NOCM reaction cycle, while O_2_1 refers to the catalyst obtained after the O_2 regeneration of CH_4_1. The Pt/CeO_2 catalyst after the long-term test is denoted by CH_4_used, referring to the catalyst used for 5 h NOCM reaction.

Supplementary figures and tables

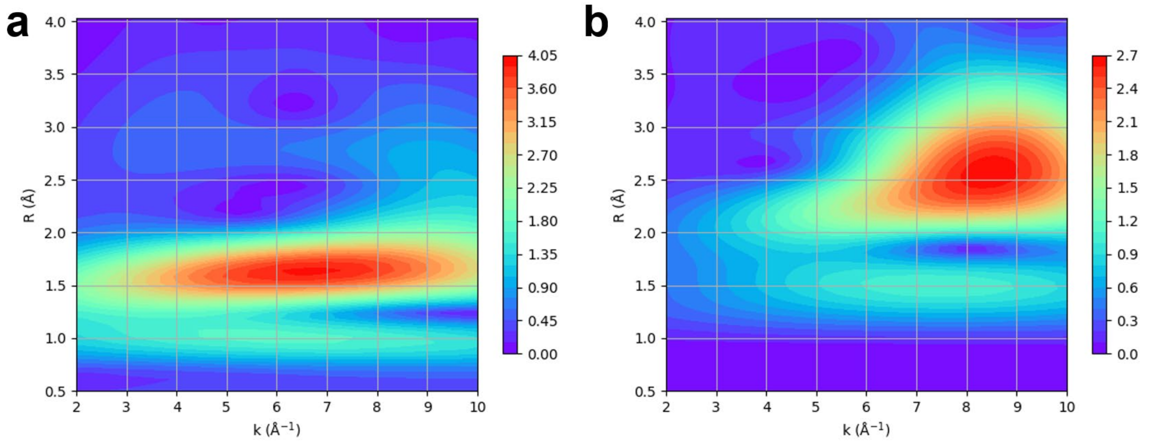

Figure S1. Wavelet transformed (WT) k^3-weighted EXAFS measured at the Pt L_3 edge of the (a) fresh and (b) used Pt/CeO_2 catalyst after 5 h of NOCM reaction. The Morlet mother wavelet was used with the parameters of $\sigma = 1$ and $\eta = 10$. Obvious difference can be observed between the fresh and used catalysts. An additional shell with WT maxima of 2.5 Å and 8.5 $Å^{-1}$ appeared after the reaction, suggesting the bonding between Pt and the atoms heavier than oxygen. However, the actual phase cannot be determined based on this WT EXAFS analysis.

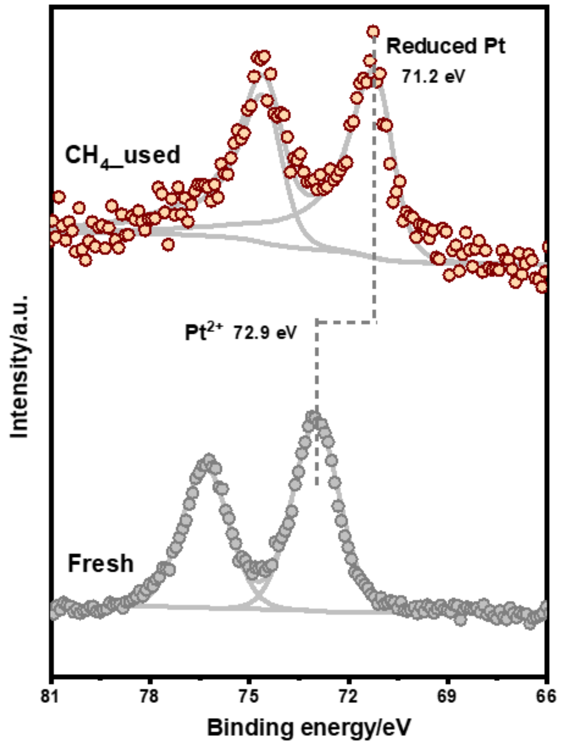

Figure S2. Pt *4f* XPS results of fresh and used Pt/CeO_2 catalysts.

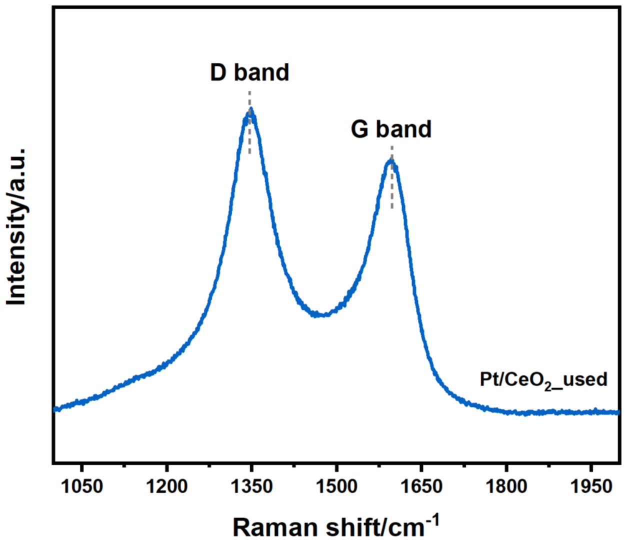

Figure S3. Raman analysis of used Pt/CeO_2 catalyst. The reaction was carried out at 800 °C under 10 mL/min 95 vol.% CH_4 with 5 vol.% Ar for 300 min.

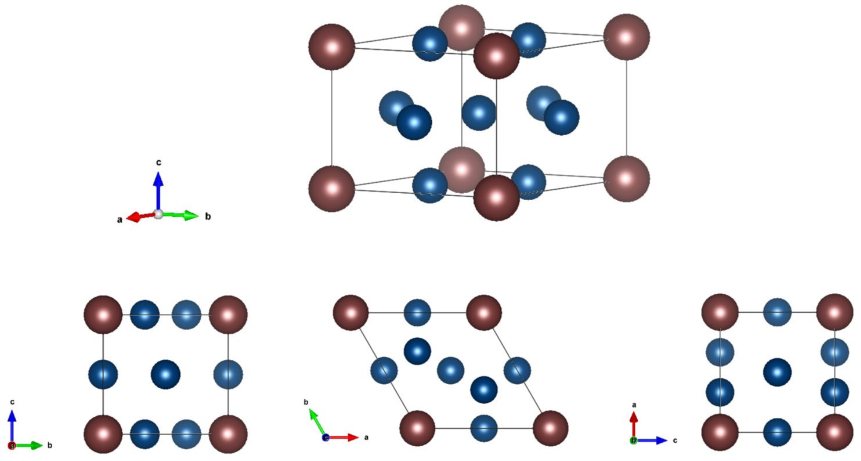

Figure S4. Crystal structure of $CePt_5$. Space group: P6/mmm. The crystal data were retrieved from the Materials Project for $CePt_5$ (mp-542777) from database version v2022.10.28.[10] Brown: Ce; blue: Pt.

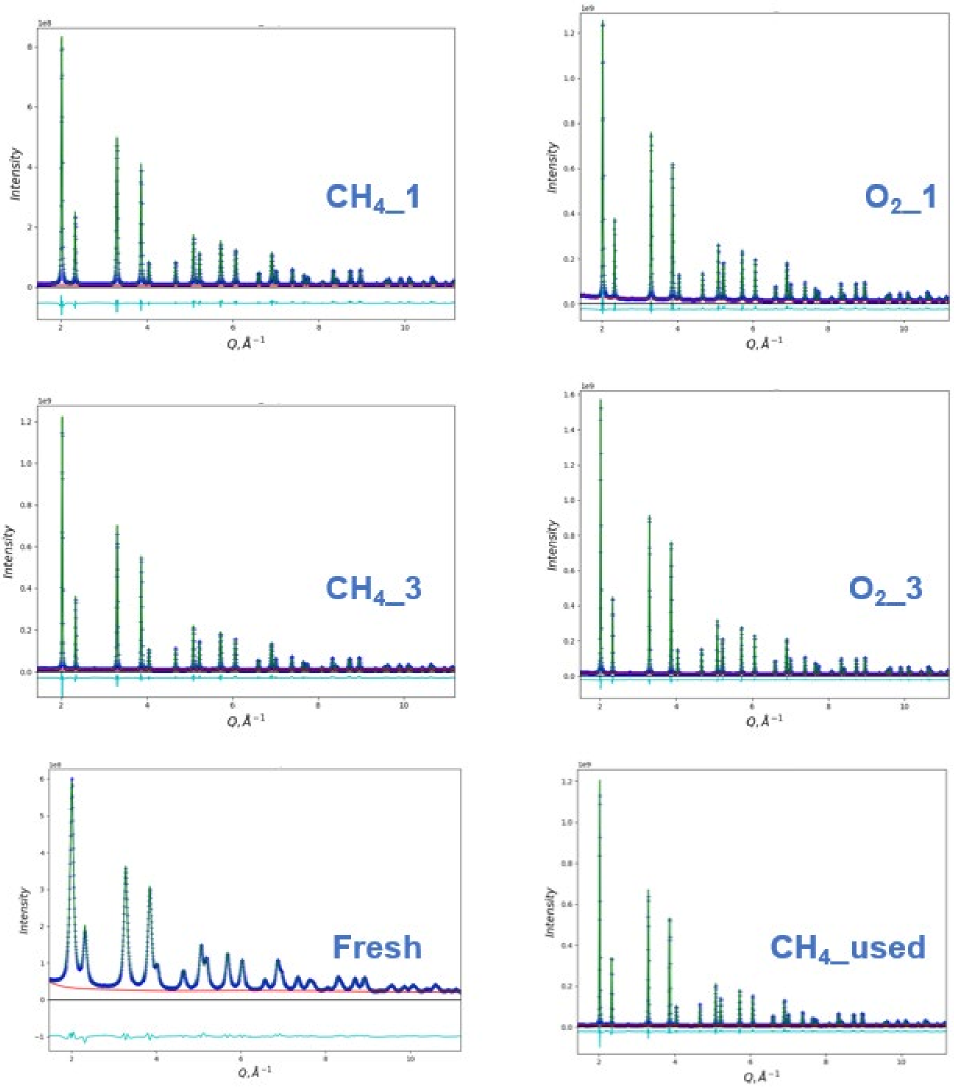

Figure S5. Rietveld refinement of XRD patterns of Pt/CeO_2 in the fresh state and after reaction and regeneration treatments. The raw data are shown in blue "+", the fitted results are displayed using green lines, the background and the difference between the raw data and the fitted curves are shown in red and cyan lines, respectively.

Table S1. Rietveld refinement of XRD patterns of Pt/CeO_2 in the fresh state and after reaction and regeneration cycles.

Sample	CeO_2 particle size/nm	Ce_2O_3 particle size/nm	Pt^0 particle size/nm	$CePt_5$ particle size/nm	CeO_2 cell parameter/Å	*wR*/%
Fresh	7	-	-	-	5.41	3.5
CH_4_used	265	16	-	7	5.38	7.8
CH_4_1	40	11	-	4	5.38	6.9
O_2_1	56	-	17	-	5.39	5.0
CH_4_3	120	14	-	8	5.39	7.4
O_2_3	117	-	19	-	5.38	4.4

The CeO_2 particle size and unit cell parameter were obtained through Rietveld refinement analysis, and the particle sizes of Ce_2O_3, Pt^0, and $CePt_5$ were determined using the Scherrer equation.

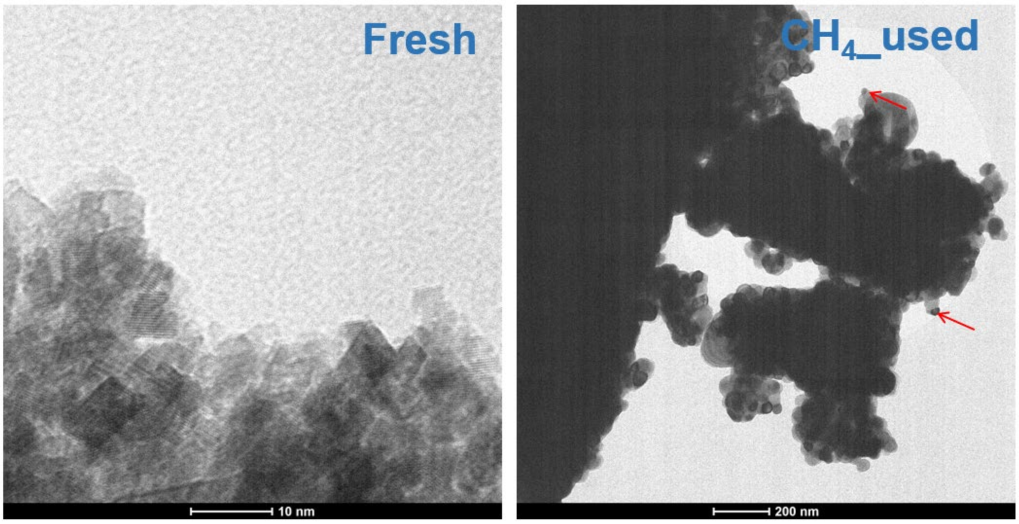

Figure S6. TEM images of fresh and used Pt/CeO_2 catalysts after 5 h of NOCM reaction. The red arrows may indicate the positions of Pt particles based on the contrast.

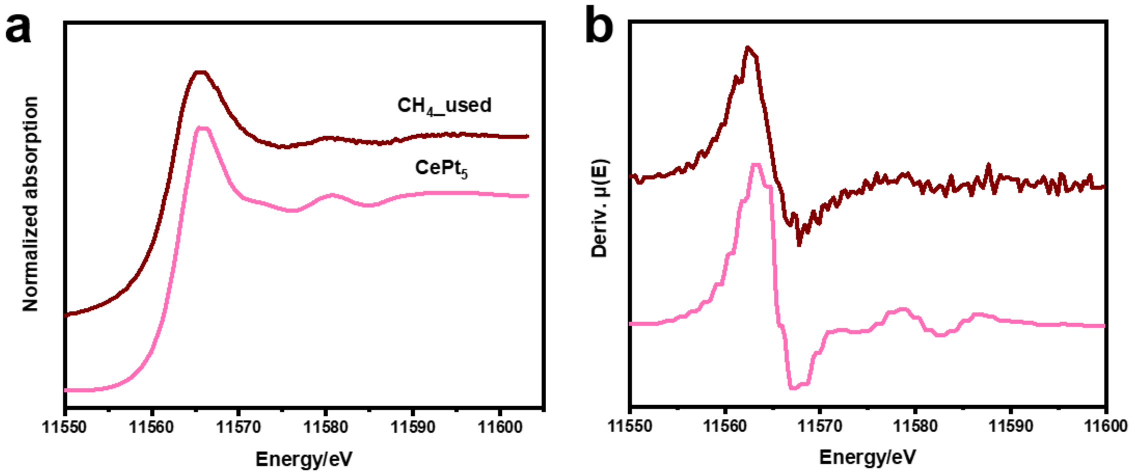

Figure S7. Comparison of (a) XANES of the Pt/CeO_2 catalyst after 5 h NOCM reaction and a simulated XANES of $CePt_5$ at the Pt L_3 edge, and (b) their first derivatives.

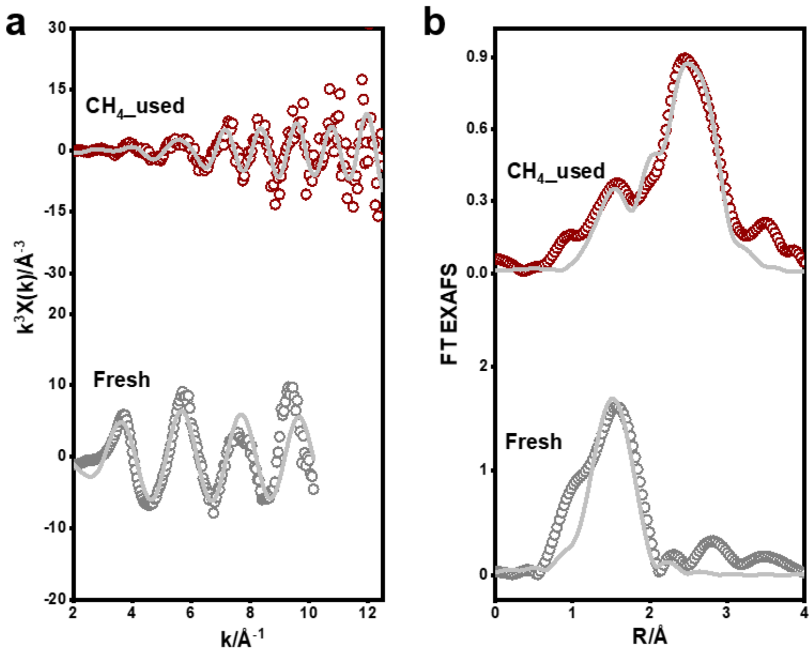

Figure S8. EXAFS fits of Pt/CeO_2 in the fresh state and after 5 h NOCM reaction in (a) *k*-space and (b) *R*-space. The open circles display the raw data and the fitting results are shown in grey lines. The spectra were collected at the Pt L_3 edge. The scattering paths obtained from $CePt_5$ were used to fit the EXAFS spectra of the samples after NOCM reaction. The scattering path from PtO_2 was used to fit the Pt-O shell. Reasonable fitting can be obtained by using the above models. Therefore, the formation of $CePt_5$ phase during NOCM reaction can be supported by EXAFS.

Table S2. EXAFS fitting results of fresh and used Pt/CeO_2 catalysts

Sample	Path	CN	$\sigma^2/Å^2$	ΔE_0/eV	R/Å	R-factor
CH_4_used	Pt-O	0.8	0.0006	1.1	1.93	0.049
	Pt-Pt	6.4	0.0044		2.68	
	Pt-Ce	2.9	0.0595		3.02	
	Pt-Pt	2.4	0.0112		3.07	
Fresh	Pt-O	3.8	0.0013	11.9	1.96	0.038

The amplitude reduction factor (S_0^2) was fixed at 0.925; CN: coordination number; σ^2: Debye-Waller factor; ΔE_0: energy shift; *R*: distance between absorbing atom and scattering atom.

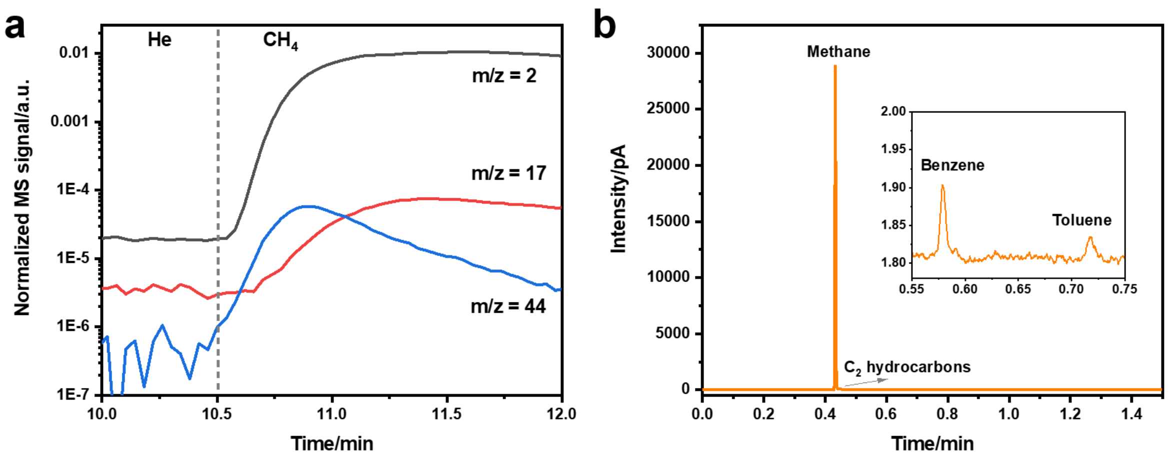

Figure S9. (a) MS data of m/z = 2 (H_2), m/z = 17 (H_2O), and m/z = 44 (CO_2) signals during a switch from He to CH_4. The NOCM reaction was performed at 800 °C using 100 mg of catalyst under 10 mL/min of 95 vol.% CH_4 and 5 vol.% Ar. (b) GC-FID chromatogram recorded after 40 min reaction during the long-term activity measurement. Inset shows the peaks of benzene and toluene. The NOCM reaction was performed at 800 °C under 10 mL/min of 95 vol.% CH_4 and 5 vol.% Ar using 100 mg of catalyst.

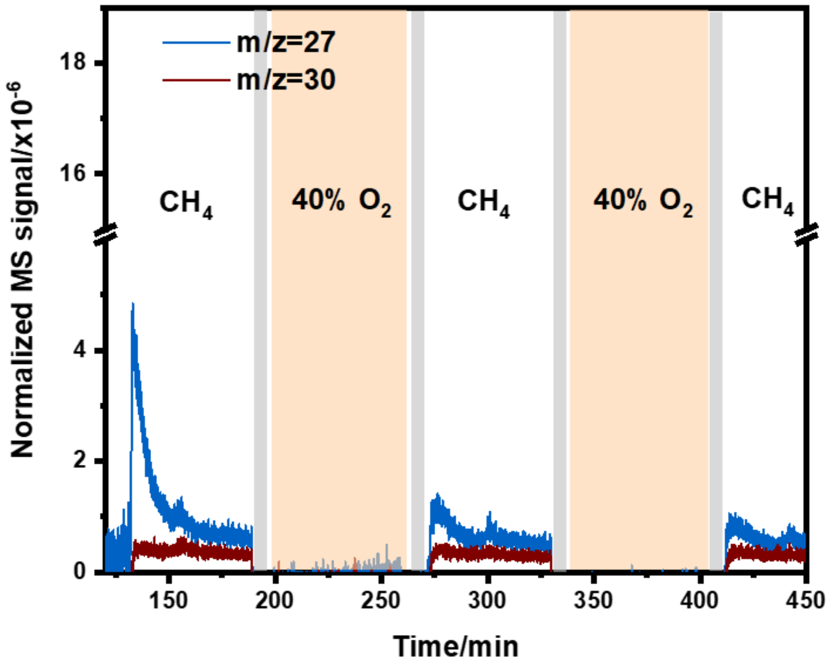

Figure S10. Reaction-regeneration experiments using 100 mg of CeO_2 at 800 °C. A flow of 50 mL/min of He was used to purge the reactor for 10 min between the NOCM reaction in 10 mL/min of 95 vol.% CH_4 and the regeneration in a flow of 10 ml/min of 40 vol.% O_2. The gray and orange zones indicate the He-purging and the regeneration in O_2, respectively.

Table S3. Surface fraction of Pt^{2+} and $Pt^0/CePt_5$ in fresh and used catalysts.

Catalyst	Fraction/at.%		Pt/Ce
	Pt^{2+}	Reduced Pt	
Fresh	100	0	1.5
CH_4_1	25	75	4.2
O_2_1	88	12	3.9
CH_4_3	0	100	1.4
O_2_3	76	24	3.1

The fractions of Pt^{2+} and reduced Pt species are obtained from fits of the Pt *4f* XPS results. Reduced Pt species refer to either Pt in $CePt_5$ and Pt^0, as it is not possible to distinguish these two species by XPS. The Pt/Ce atomic ratio is obtained from XPS survey spectra. The increased Pt/Ce atomic ratio of CH_4_1 compared with the fresh catalyst suggests the extraction of Pt from the bulk of Pt/CeO_2 catalyst during reaction.

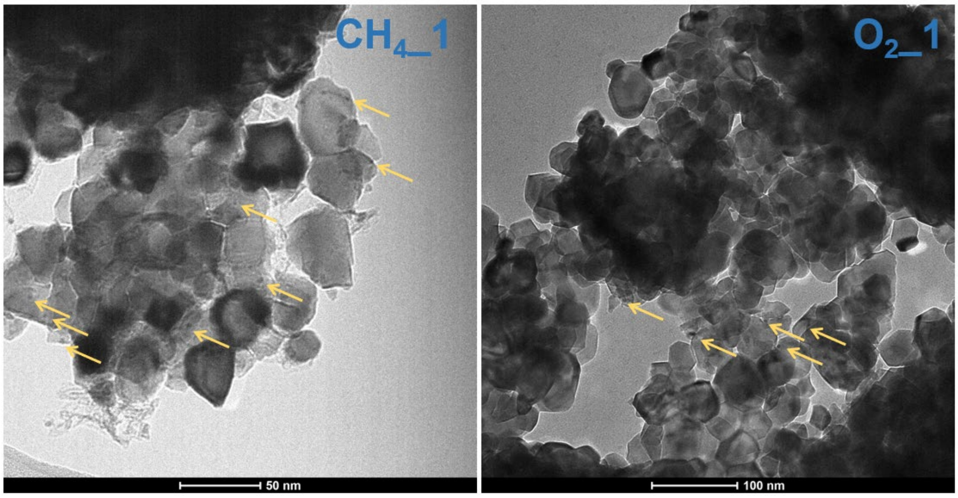

Figure S11. TEM images of Pt/CeO_2 catalysts after the first NOCM reaction and after the first O_2 regeneration treatment. Pt-containing particles on the CeO_2 support are indicated by yellow arrows. Therefore, the agglomerated Pt species are only partially redispersed after the regeneration in O_2.

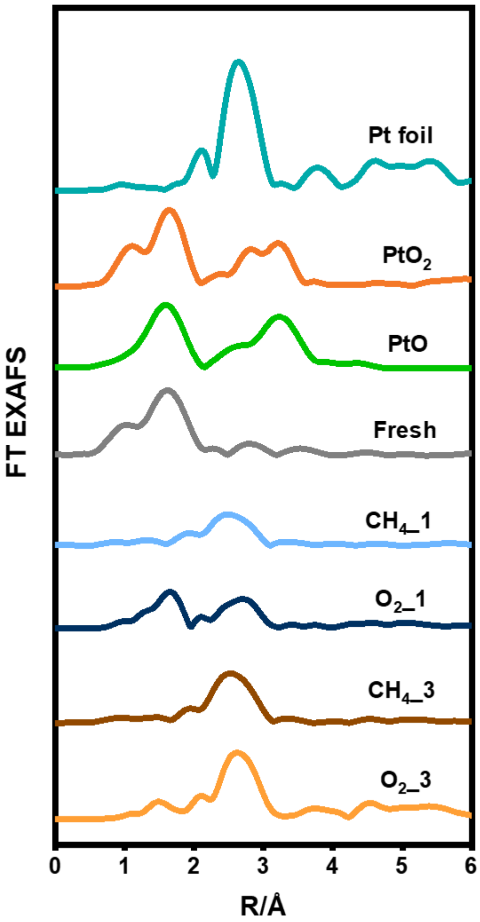

Figure S12. k^3-weighted FT EXAFS of fresh and used Pt/CeO_2 catalysts including Pt foil and PtO_2 references. The spectra were collected at the Pt L_3 edge. No coordination shell related to Pt-Pt scattering paths is observed for the fresh Pt/CeO_2 catalyst compared with the Pt foil reference. The coordination shell related to the Pt-O single scattering path can be seen after the O_2 regeneration, and it becomes less pronounced after the third regeneration treatment compared with the first one.

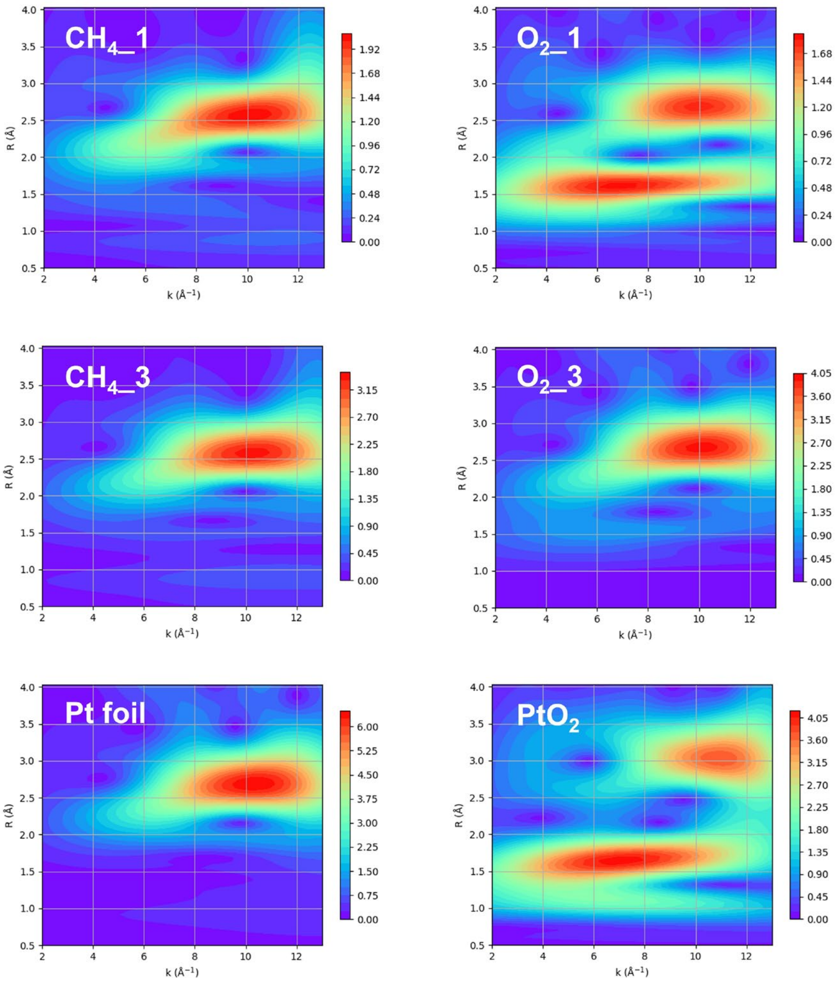

Figure S13. Wavelet transformed quasi *in situ* EXAFS of Pt/CeO_2 catalysts at different reaction-regeneration stages and the related references. The spectra were collected at the Pt L_3 edge. The k^3-weighted EXAFS was used with the Morlet mother wavelet using the parameters of σ = 1 and η = 10.

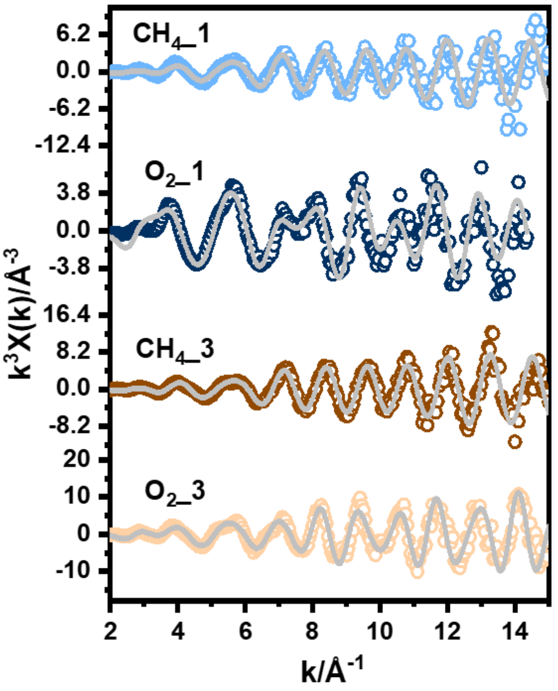

Figure S14. The *k*-space EXAFS data and the corresponding fitting results obtained through quasi *in situ* EXAFS analysis. The data were collected at the Pt L_3 edge. The open circles represent the raw data, while the fitting results are indicated by the gray lines. The scattering paths obtained from Pt metal were used in the fitting of the samples after O_2 regeneration, while the paths from $CePt_5$ were used to fit the EXAFS spectra of the samples after NOCM reaction. The Pt-O scattering path extracted from the PtO_2 model was used to fit the Pt-O shell.

Table S4. EXAFS fitting results of the Pt/CeO_2 catalysts after different reaction/regeneration treatments.

Sample	Path	CN	σ^2/Å^2	ΔE_0/eV	R/Å	R-factor
CH_4_1	Pt-O	0.4	0.0034	-0.1	1.90	0.034
	Pt-Pt	4.4	0.0048		2.68	
	Pt-Ce	2.0	0.0199		3.31	
	Pt-Pt	0.7	0.0102		3.23	
O_2_1	Pt-O	2.8	0.0038	9.3	1.97	0.044
	Pt-Pt	3.6	0.0052		2.76	
	Pt-Pt	2.4	0.0117		3.87	
CH_4_3	Pt-O	0.3	0.0015	1.7	1.92	0.010
	Pt-Pt	6.0	0.0048		2.68	
	Pt-Ce	3.2	0.0750		3.01	
	Pt-Pt	2.8	0.0560		3.02	
O_2_3	Pt-O	1.0	0.0020	7.6	1.95	0.025
	Pt-Pt	8.0	0.0050		2.75	
	Pt-Pt	3.5	0.0084		3.88	

The amplitude reduction factor (S_0^2) was fixed at 0.925; *CN*: coordination number; σ^2: Debye-Waller factor; ΔE_0: energy shift; *R*: distance between absorbing atom and scattering atom. The Pt-O single scattering path was obtained from the PtO_2 structure. The scattering paths obtained from the Pt^0 structure are used in the EXAFS fitting of samples after the O_2 regeneration, and the paths from $CePt_5$ are employed in the fitting of the samples after NOCM reaction in CH_4. Reasonable EXAFS fitting quality can be achieved, suggesting the presence of the above structures in catalysts. The increased coordination number in the second shell can be seen by comparing CH_4_1 and CH_4_3 (and O_2_1 and O_2_3), which indicates the growth of Pt^0 and $CePt_5$ particles upon CH_4 and O_2 treatments.

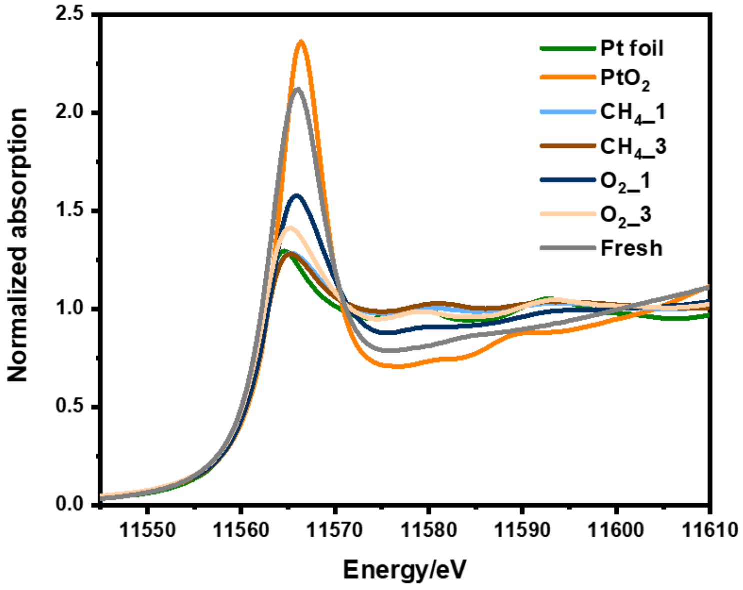

Figure S15. Pt L_3-edge XANES spectra collected in total fluorescence yield mode of fresh and used Pt/CeO_2 catalysts. The Pt foil and PtO_2 reference are also included for comparison. The white line intensity increases after the regeneration in O_2, and decreased after the NOCM reaction in CH_4. Since the redispersion of Pt species can be observed upon O_2 regeneration, the white line intensity can be used to track the sintering and redispersion of Pt sites.

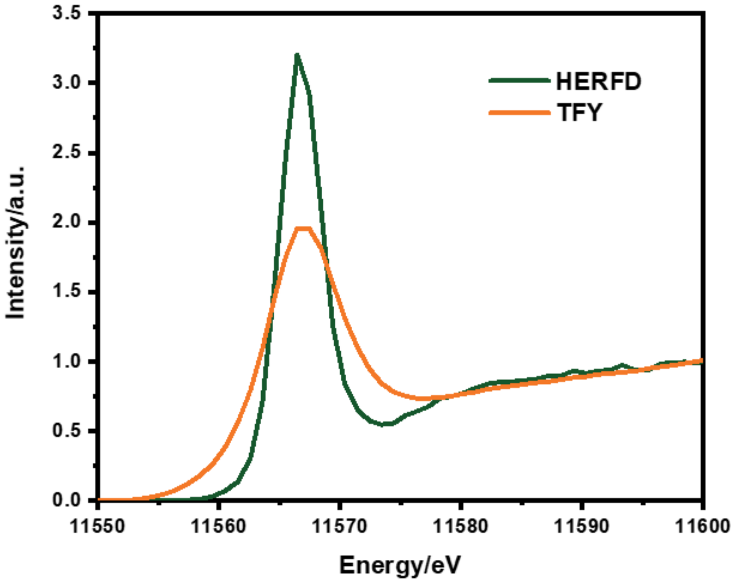

Figure S16. Pt L_3-edge XANES spectra of PtO_2 recorded in HERFD mode and total fluorescence yield (TFY) mode simultaneously at ID26 beamline of ESRF.

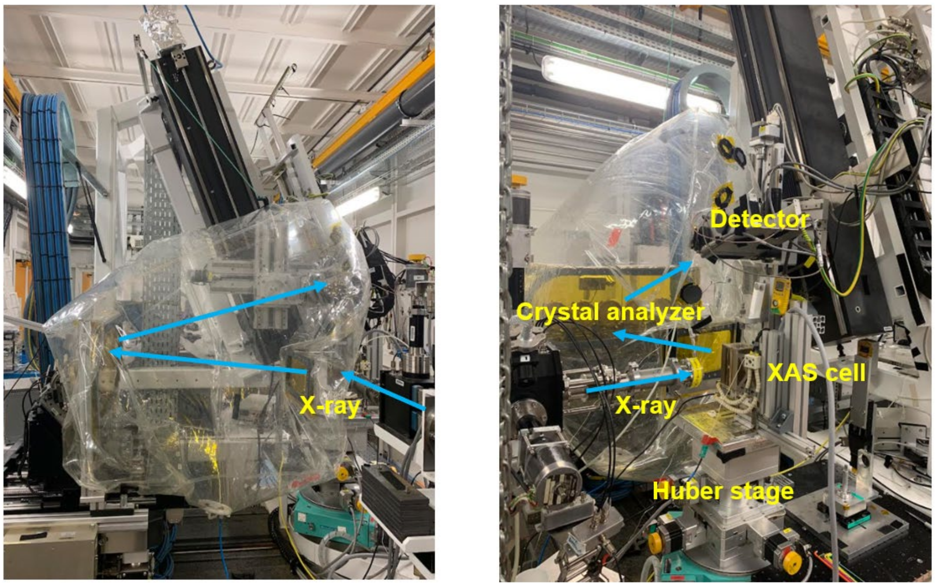

Figure S17. The home-built high-temperature *in situ* XAS cell adapted to the Huber stage at ID26 beamline of ESRF.[11, 12] The crystal analyzers were not installed when taking the pictures. A helium bag was used to reduce the X-ray absorption by air.

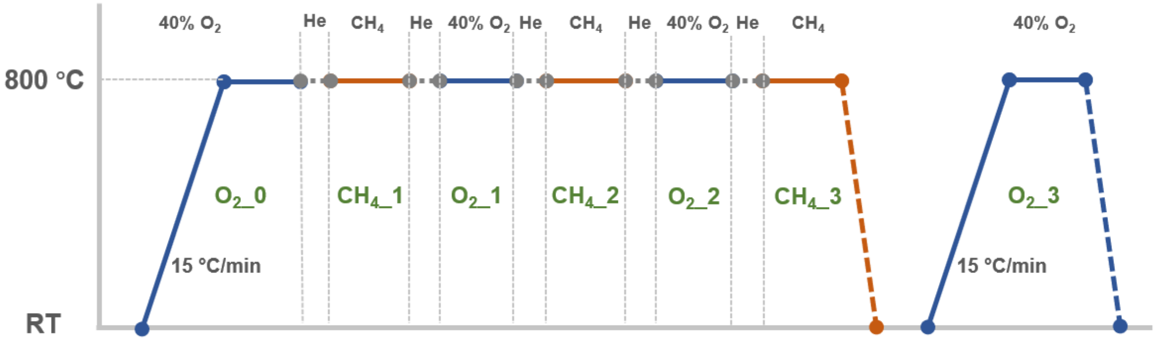

Figure S18. Experimental procedure during *in situ* HERFD-XANES measurements.

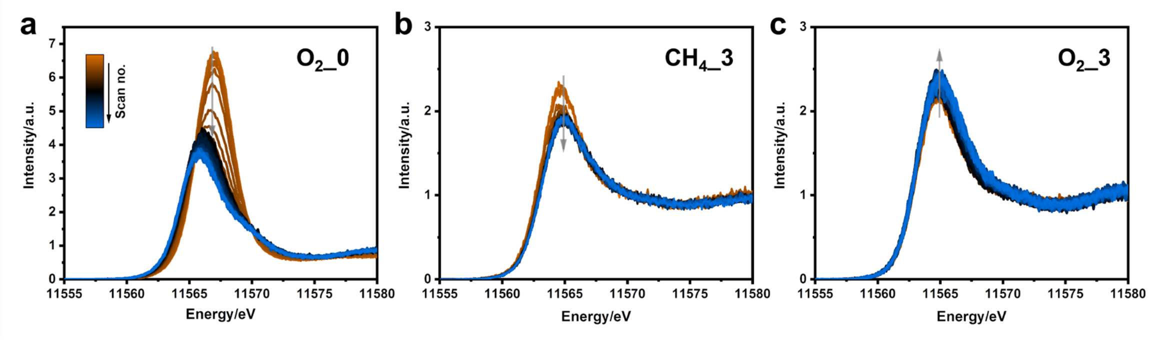

Figure S19. *In situ* HERFD-XANES spectra recorded at the Pt L_3 edge during the O_2_0, CH_4_3, and O_2_3 periods.

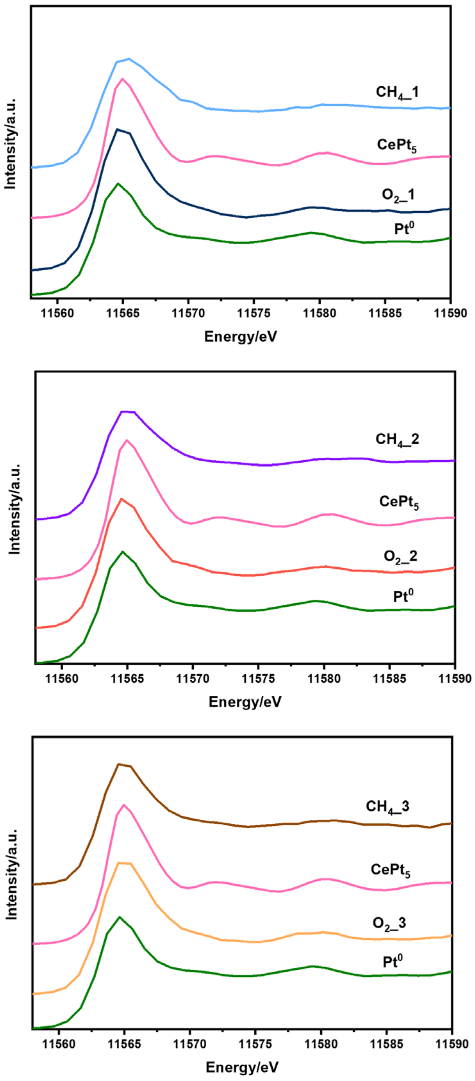

Figure S20. Pt L_3-edge HERFD-XANES spectra collected at different reaction-regeneration stages. The spectra were recorded when the state of Pt was stabilized during the measurements. The Pt foil and $CePt_5$ references are also included for comparison, in which the HERFD-XANES of $CePt_5$ at the Pt L_3 edge was obtained by the simulation using FEFF10.

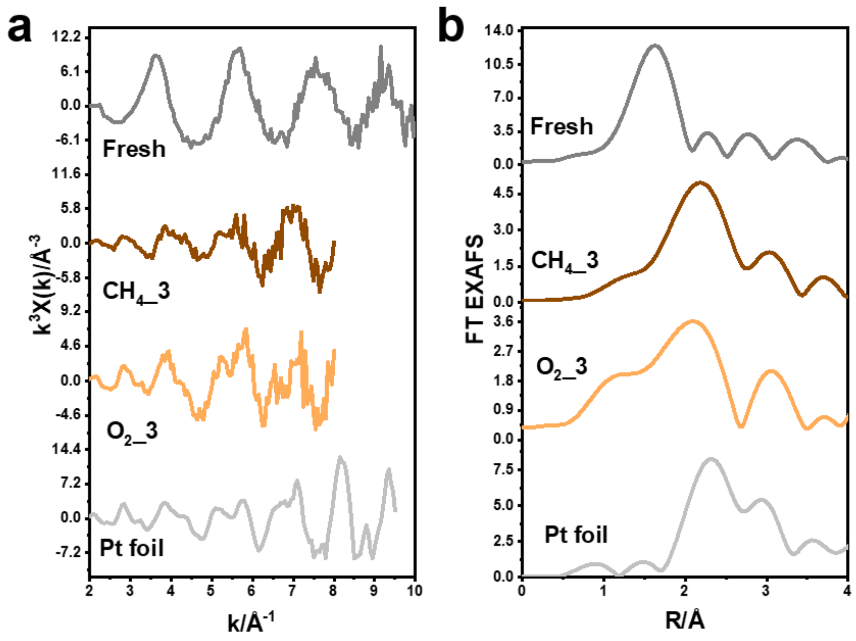

Figure S21. (a) The *k*-space data of the HERFD-EXAFS and (b) k^3-weighted Fourier transformed HERFD-EXAFS spectra. The HERFD-EXAFS data were recorded at room temperature. The data were collected at the Pt L_3 edge. The EXAFS fitting analysis was not carried out due to the limited signal to noise ratio. However, the data show an additional shell related to a Pt-O single scattering path in O_2_3.

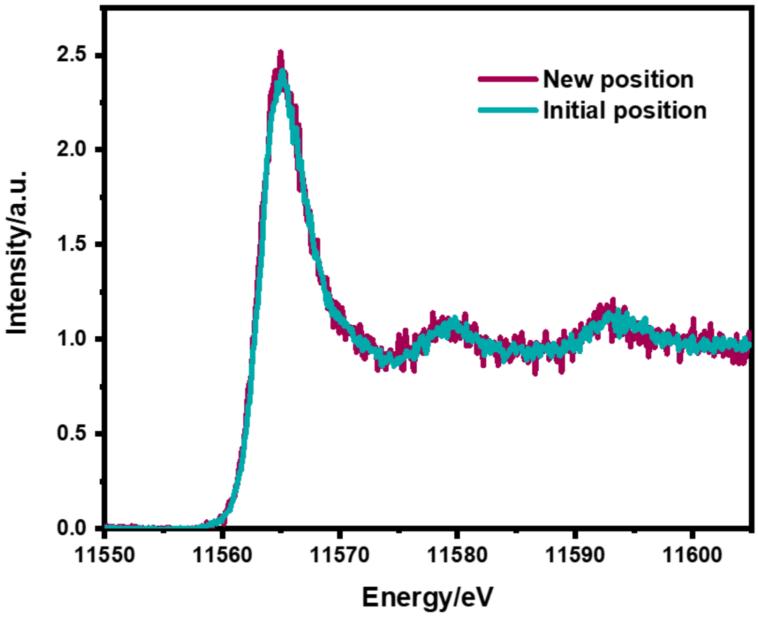

Figure S22. Pt L_3-edge HERFD-XANES collected after *in situ* tests at two positions in the isothermal zone of the reactor. The X-ray beam was focused on the initial position for the whole *in situ* measurements. No obvious difference can be seen, suggesting there was no beam damage.

References

(1) Pereira-Hernández, X. I.; DeLaRiva, A.; Muravev, V.; Kunwar, D.; Xiong, H.; Sudduth, B.; Engelhard, M.; Kovarik, L.; Hensen, E. J. M.; Wang, Y.; Datye, A. K., Tuning Pt-CeO_2 interactions by high-temperature vapor-phase synthesis for improved reducibility of lattice oxygen. *Nat. Commun.* **2019,** *10*, 1358.

(2) Kieffer, J.; Valls, V.; Blanc, N.; Hennig, C., New tools for calibrating diffraction setups. *J. Synchrotron Radiat.* **2020,** *27*, 558-566.

(3) Toby, B. H.; Von Dreele, R. B., GSAS-II: the genesis of a modern open-source all purpose crystallography software package. *J. Appl. Crystallogr.* **2013,** *46*, 544-549.

(4) Newville, M., Larch: An analysis package for XAFS and related spectroscopies. *J. Phys. Conf. Ser.* **2013,** *430*, 012007.

(5) Kas, J. J.; Vila, F. D.; Pemmaraju, C. D.; Tan, T. S.; Rehr, J. J., Advanced calculations of X-ray spectroscopies with FEFF10 and Corvus. *J. Synchrotron Radiat.* **2021,** *28*, 1801-1810.

(6) Rehr, J. J.; Kas, J. J.; Vila, F. D.; Prange, M. P.; Jorissen, K., Parameter-free calculations of X-ray spectra with FEFF9. *Phys. Chem. Chem. Phys.* **2010,** *12*, 5503-5513.

(7) Ankudinov, A. L.; Rehr, J. J.; Low, J. J.; Bare, S. R., Theoretical interpretation of XAFS and XANES in Pt clusters. *Top. Catal.* **2002,** *18*, 3-7.

(8) Safonova, O. V.; Tromp, M.; van Bokhoven, J. A.; de Groot, F. M. F.; Evans, J.; Glatzel, P., Identification of CO adsorption sites in supported Pt catalysts using high-energy-resolution fluorescence detection X-ray spectroscopy. *J. Phys. Chem. B* **2006,** *110*, 16162-16164.

(9) Solé, V. A.; Papillon, E.; Cotte, M.; Walter, P.; Susini, J., A multiplatform code for the analysis of energy-dispersive X-ray fluorescence spectra. *Spectrochim. Acta B: At. Spectrosc.* **2007,** *62*, 63-68.

(10) Jain, A.; Ong, S. P.; Hautier, G.; Chen, W.; Richards, W. D.; Dacek, S.; Cholia, S.; Gunter, D.; Skinner, D.; Ceder, G.; Persson, K. A., Commentary: The Materials Project: a materials genome approach to accelerating materials innovation. *APL Mater.* **2013,** *1*, 011002.

(11) Glatzel, P.; Harris, A.; Marion, P.; Sikora, M.; Weng, T.-C.; Guilloud, C.; Lafuerza, S.; Rovezzi, M.; Detlefs, B.; Ducotte, L., The five-analyzer point-to-point scanning crystal spectrometer at ESRF ID26. *J. Synchrotron Radiat.* **2021,** *28*, 362-371.

(12) Ducotté, L.; Glatzel, P.; Marion, P.; Lapras, C.; Lesourd, M.; Harris, A.; Heyman, C., Mechanical aspects of the ID26 emission spectrometer II: improving stability for a large instrument by the use of multiple air pad supports. *Diam. Light Source Proc.* **2010,** *1*, e26.
